# Supplementary material for: A quantitative model for human neurovascular coupling with translated mechanisms from animals
Source: PLoS Comput Biol. 2023 Jan 6;19(1):e1010818. doi: 10.1371/journal.pcbi.1010818 (PMC9821752; doi:10.1371/journal.pcbi.1010818)
Supplement: S1 Appendix — (DOCX) [file pcbi.1010818.s001.docx]

The supplementary material contains the following: 1) the posterior probability profiles of the parameters. 2) Formulations of the qualitative demands used to promote the vasoactive control of the vascular smooth muscle cells (VSM), the hemoglobin (Hb) behavior, and the behavior of the local field potential (LFP) signal.

**1 Posterior probability profiles of the model parameters and model estimation**

- 1. **Posterior probability profiles for model estimation in Fig 3**

All parameter boundary values used for the generation of the posterior probability profiles, S1 Fig, are given in log10-space for all 37 available parameters in the model in S1 Table.

**1.2 Allowing the stiffness and viscoelasticity parameters to change between short and long stimulation in Fig 3**

All parameter boundary values used for the generation of the posterior probability profiles, S3 Fig, are given in log10-space for all 41 available parameters in the model in S2 Table.

**1.3 Model estimation in previous work**

Recreation of results from previous work by [1], showing that the new expanded model preserves the ability to explain pharmacological perturbations including the effect of anesthetics.

**1.4 Posterior probability profiles for model estimation in Fig 5**

All parameter boundary values used for the generation of the posterior probability profiles, S5 Fig, are given in log10-space for all 43 available parameters in the model in S3 Table.

**1.5 Posterior probability profiles for model estimation in Fig 6**

All parameter boundary values used for the generation of the posterior probability profiles, S6 Fig, are given in log10-space for all 52 available parameters in the model in S4 Table.

**1.6 Posterior probability profiles for model estimation in Fig 7**

All parameter boundary values used for the generation of the posterior probability profiles, S7 Fig, are given in log10-space for all 57 available parameters in the model in S5 Table.

**1.7 Comparison of posterior probability profiles**

In this section, an analysis will be given of the distributions of the posterior probability profiles generated for the different studies.

**1.7.1 Mice studies**

Two of the studies, Drew et al and Desjardin et al [2,3], were carried out on mice. The model was fitted separately to the experimental data from these studies. Therefore, it is of interest to observe how the posterior probability profiles, S1 and S5 Figs, compare to each other and if the model identifies similar behavior between them.

Starting with the stimulus scaling parameters, k_u1-3_ from the Drew study and k_u1-3_ Sens Long parameters from the Desjardins study (the experimental setup for these is the most similar to each other). These parameters were included in the model as it is not known how the stimulus paradigm is translated between experiments, different subjects, and studies. Following this, one can see that these profiles do differ from each other, which is expected from the model design. Correspondingly, we can also observe that the stimulus scaling parameters do differ between the experimental setups within the Desjardins study, S5 Fig (optogenetic inhibitory, OGin, optogenetic excitatory, OGex, sensory long, Sens Long, and sensory short, Sens Short). More interestingly, one can see that the model has chosen quite similar profiles for the two sets of neuronal signaling parameters, KPF, KIN, and KINF, and elimination rate parameters, Sink_NO, NPY, PYR_, S1 and S5 Figs. These profiles indicate that the model identifies that similar neuronal behavior is needed to generate the vascular changes present in the two studies (vessel diameter change and hemoglobin changes). Likewise, most of the posterior probability profiles of the intracellular signaling parameters are similar between the studies.

Continuing, the profiles of the vascular parameters, the viscoelasticity (vis1-3) and stiffness coefficients (K1-K3), have similar distributions. Although, these parameters have complementary effects to each other and do not hold the whole model behavior by themselves. Overall, the model identifies similar profiles between the different independently fitted studies – indicating that the model finds a similar behavior in both data sets.

**1.7.2 All studies**

The four probability posterior profiles, S1 and S5-S7 Figs, were achieved by fitting the model independently to the experimental data from the different studies, Drew et al, Desjardin et al, Shmuel et al, and Huber et al [2,4–6]. As in the case of the previous section, 1.7.1, one can observe that the different sets of stimulus scaling parameters, k_u1-3_, do vary between the posterior probability profiles. This is expected from the model design, where these parameters are translating the effect of the different stimulus paradigms to the model. The reader is pointed to Table 1 to see how the parameters were distributed in the different experimental settings and studies, but in short, the Drew study has 1 set of stimulus parameters, the Desjardins study has 4, the Shmuel study has 2, and the Huber study has 4. Moving on, the neuronal signaling parameters, KPF, KIN, and KINF, and elimination rate parameters, Sink_NO, NPY, PYR_ do share similar profiles between the four figures, S1 and S5-S7 Figs. This is quite remarkably since the only information carried over between the studies are our qualitative demands, which suggests that these demands carry enough information of the system for the model to identify a similar behavior between the species. This especially, holds for the mice profiles (S1 and S5 Figs) and human profile (S7 Fig), where the weight of the posterior probability profiles lies in the same region – although the range of distribution is wider for the human profile (S7 Fig). The wider range could be explained by the experimental stimuli being less intensive for the studies carried out on humans and monkeys (S6 and S7 Figs). The biggest outliers can be found in S6 Fig, namely parameters kPF1, kIN2, and sinkN_NO_. This could be a result of the behavior that the local field potential experimental data introduces to the model behavior.

Moreover, the profiles for the intracellular signaling parameters and the vascular parameters, the viscoelasticity (vis1-3), and stiffness coefficients (K1-K3), follow this general trend as well. Not all parameters fall into the same distribution, remarkably many do have similar ranges and distribution weights. In the case of the negative parameters, S6 and S7 Figs, one can observe that some of them i.e., kIN2, kINF1, kINF2, and sinkN_NPY_, have similar profiles to the corresponding set of positive parameters, even tough this is not imposed. One could further investigate this to identify the behaviors that the model changes, and consecutively do not change, between the different stimulus settings. Overall, it is interesting to observe that the qualitative demands seem to be able to preserve enough information about the experimental system for similar posterior probability profiles to arise in sequential analysis. This preservation of behavior argues for the behavior in the different species is similar enough for the model to be able to describe the different experimental data with similar behavior.

**References**

1. Sten S, Elinder F, Cedersund G, Engström M. A quantitative analysis of cell-specific contributions and the role of anesthetics to the neurovascular coupling. Neuroimage. 2020 Jul 15;215:116827.

2. Drew PJ, Shih AY, Kleinfeld D. Fluctuating and sensory-induced vasodynamics in rodent cortex extend arteriole capacity. Proc Natl Acad Sci [Internet]. 2011 May 17;108(20):8473–8. Available from: http://www.pnas.org/cgi/doi/10.1073/pnas.1100428108

3. Desjardins M, Kılıç K, Thunemann M, Mateo C, Holland D, Ferri CGL, et al. Awake Mouse Imaging: From Two-Photon Microscopy to Blood Oxygen Level–Dependent Functional Magnetic Resonance Imaging. Biol Psychiatry Cogn Neurosci Neuroimaging [Internet]. 2019 Jun;4(6):533–42. Available from: https://linkinghub.elsevier.com/retrieve/pii/S2451902218303240

4. Uhlirova H, Kılıç K, Tian P, Thunemann M, Desjardins M, Saisan PA, et al. Cell type specificity of neurovascular coupling in cerebral cortex. Elife [Internet]. 2016 May 31 [cited 2021 Jan 27];5:e14315. Available from: https://elifesciences.org/articles/14315

5. Shmuel A, Augath M, Oeltermann A, Logothetis NK. Negative functional MRI response correlates with decreases in neuronal activity in monkey visual area V1. Nat Neurosci [Internet]. 2006 Apr 19;9(4):569–77. Available from: http://www.nature.com/articles/nn1675

6. Huber L, Goense J, Kennerley AJ, Ivanov D, Krieger SN, Lepsien J, et al. Investigation of the neurovascular coupling in positive and negative BOLD responses in human brain at 7T. Neuroimage [Internet]. 2014 Aug;97:349–62. Available from: https://linkinghub.elsevier.com/retrieve/pii/S1053811914002778
