# Supplementary material for: A quantitative model for human neurovascular coupling with translated mechanisms from animals
Source: PLoS Comput Biol. 2023 Jan 6;19(1):e1010818. doi: 10.1371/journal.pcbi.1010818 (PMC9821752; doi:10.1371/journal.pcbi.1010818)
Supplement: S2 Appendix — (DOCX) [file pcbi.1010818.s002.docx]

**S2 Qualitative demands during simulation**

**S2.1 Order of the three signaling arms**

**S2.1.1 Positive stimulation**

Following qualitative demands were used to promote the VSM behavior:

$NOVSM>PGE_{2}VSM, if t<t_{intial peak}$ (S1)

The initial peak should be produced by NO and not by the slower release of PGE_2_.

$PGE_{2}VSM>NOVSM, if t_{initial peak}<t<t_{stimulation}$ (S2)

After the initial peak, the increasing impact of PGE_2_ should dominate the dilating effect.

$-NPYVSM<PGE_{2}VSM, if t<t_{stimulation}$ (S3)

$-NPYVSM<NOVSM, if t<t_{initial peak}$ (S4)

$NPYVSM>0, if t<t_{stimulation}$ (S5)

Demands to prevent the model from using the constricting effects of NPY negatively (i.e., dilating) to replace PGE_2_ during the main dilating phase or NO during the initial peak. Additionally, NPY should not be negative during the stimulation phase.

$NPYVSM>abs\left( PGE_{2}VSM \right), if t_{post peak undershoot}<t<t_{end}$ (S6)

NPY should be the driving factor in producing the post-peak undershoot.

**S2.1.2 Negative stimulation**

$-NPYVSM>PGE_{2}VSM, if t_{post peak overshoot}<t<t_{end}$ (S7)

The post-peak overshoot should be generated by the decrease of the constricting agent NPY, rather than the decrease of PGE_2_ due to the slower nature of the NPY secretion.

**S2.2 Hb demands**

$2*abs\left( HbO \right)> abs\left( HbR \right), if t<t_{stimulation}$ (S8)

The increase of oxygenated Hb (HbO) should at least be twice the amount lost from the decrease of deoxygenated Hb (HbR).

$HbO, if t=t_{end}$ (S9)

$HbR, if t=t_{end}$ (S10)

Demands to encourage the model to try and return the Hb changes to baseline towards the end of the simulation.

**S2.3 LFP demands**

$LFP_{peak}>1.2*LFP_{plateau}$ (S11)

$LFP_{peak}>intial value of LFP_{peak}$ (S12)

The initial peak of the LFP-signal should be bigger than the value of the plateau. Additionally, the value needs to be bigger than the initial value to make sure that the LFP signal does not shrink towards a value of 0 to easier meet this demand.

$LFP_{plateau}>intial value of LFP_{plateau}$ (S13)

The value of the plateau of the LFP-signal needs to be bigger than the initial value to make sure that the LFP signal does not shrink towards a value of 0 to easier meet this demand.

$LFP_{dip}>-0.05*LFP_{peak}$ (S14)

$LFP_{dip}>intial value of LFP_{dip}$ (S15)

The dip of the LFP-signal, following the end of stimulation, should be related to the amplitude of the initial peak. Additionally, the value needs to be bigger than the initial value to make sure that the LFP signal does not shrink towards a value of 0 to easier meet this demand.
