# Supplementary figures and images for: A quantitative model for human neurovascular coupling with translated mechanisms from animals
Source: PLoS Comput Biol. 2023 Jan 6;19(1):e1010818. doi: 10.1371/journal.pcbi.1010818 (PMC9821752; doi:10.1371/journal.pcbi.1010818)

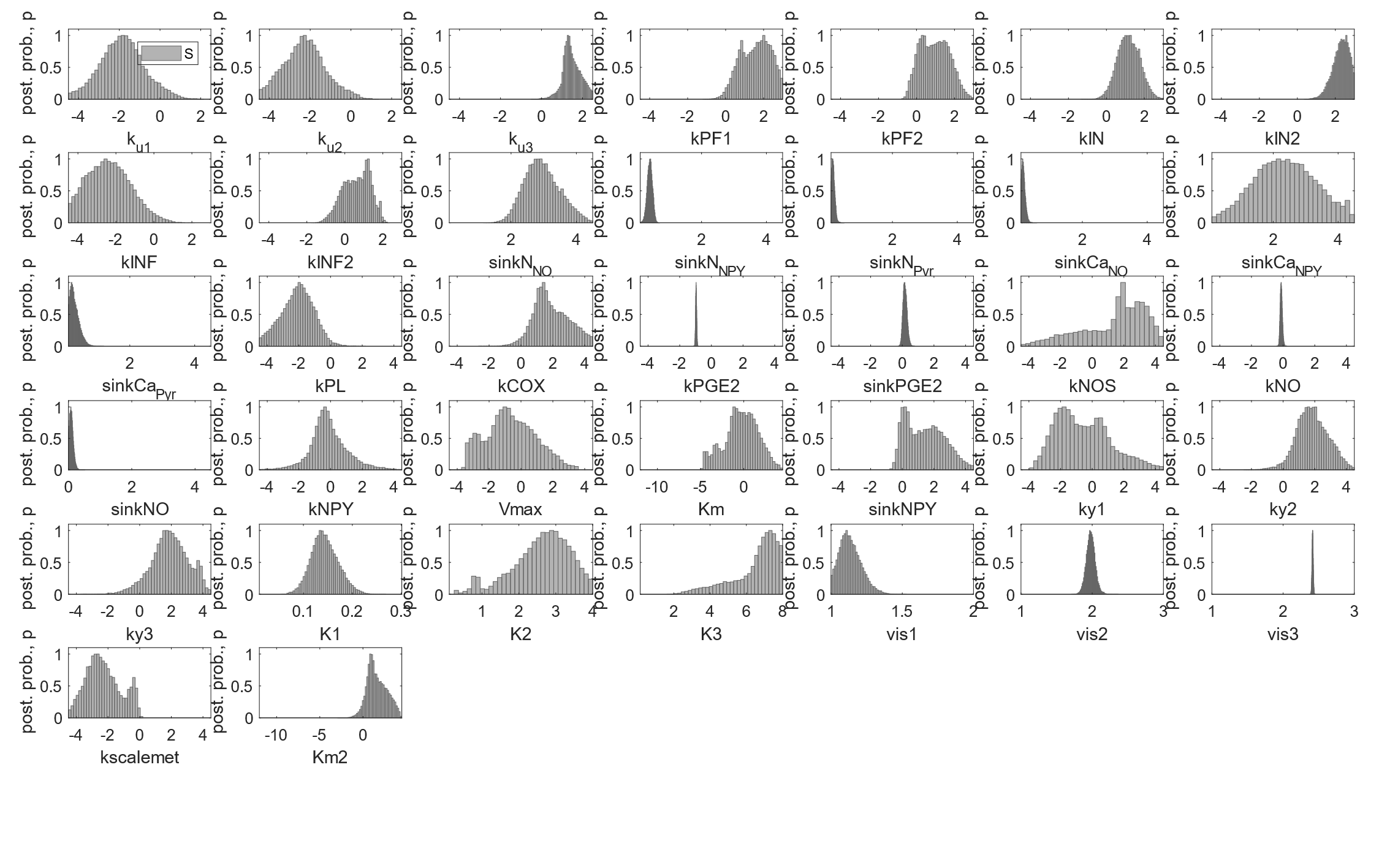

Supplement: S1 Fig — Posterior probability profile (y-axis) for each estimated model parameter (x-axis, log10 space) for the model estimated to data presented by Drew et al. [46]. (TIF) [file pcbi.1010818.s003.tif]

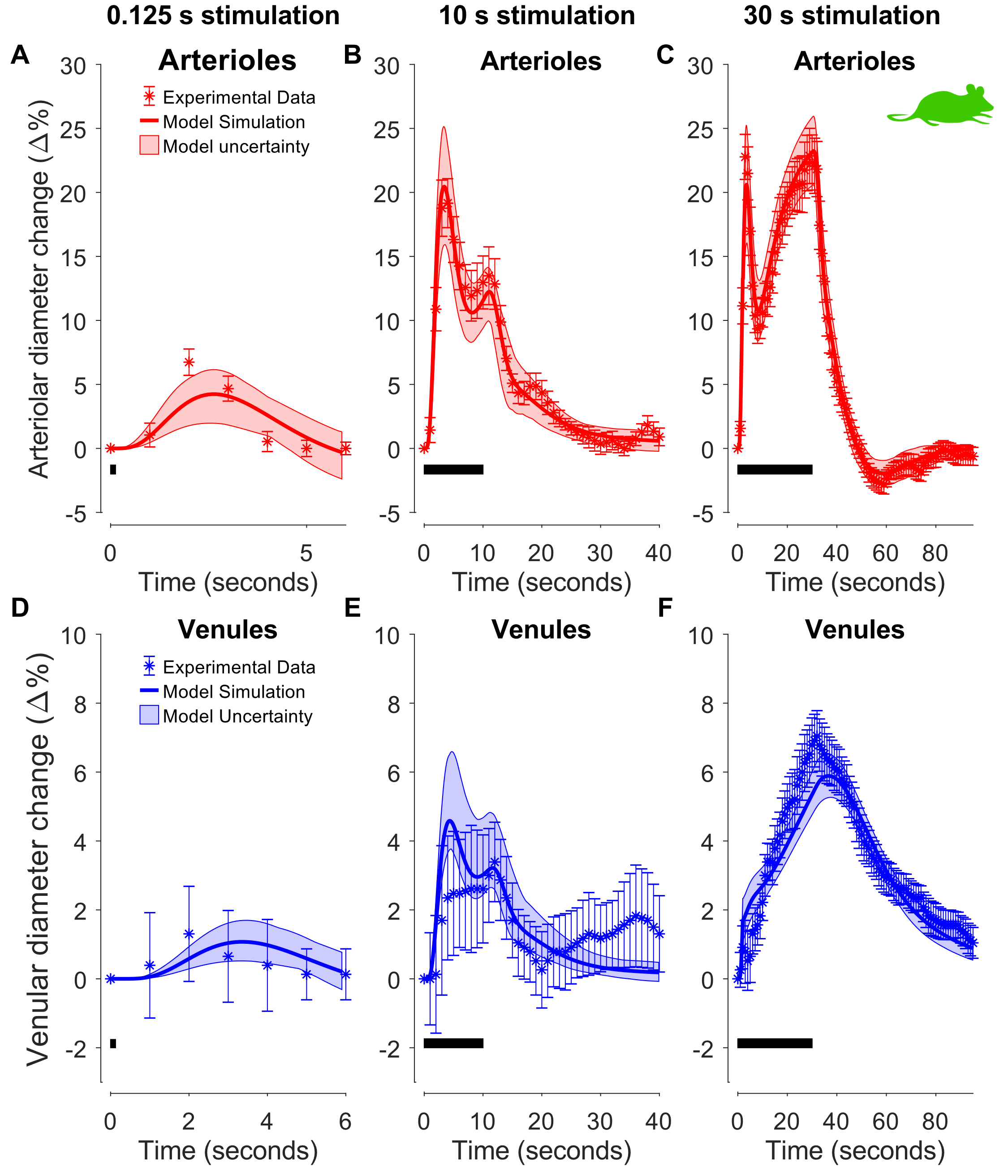

Supplement: S2 Fig — Model estimation to experimental data of arteriolar (A–C) and venular (D–F) volume changes in awake mice for three different sensory stimulation lengths: 125 ms (A & D), 10 s (B & E), and 30 s (C & F). The viscoelasticity and stiffness coefficients of the capillary and venous compartment change at t = 2s for the long stimulation (C & F). Experimental data are replotted versions of data presented in Fig 2C of the original manuscript [46]. The stimulation lengths are denoted with the black bar in the bottom left portion of each graph. For each graph: experimental data (colored symbols); The uncertainty of the experimental data is presented as SEM (colored error bars); the best model simulation is seen as a colored solid line; the model uncertainty as colored semi-transparent overlays. The x-axis represents time in seconds, and the y-axis is the normalized vessel diameter change (Δ%). (TIF) [file pcbi.1010818.s004.tif]

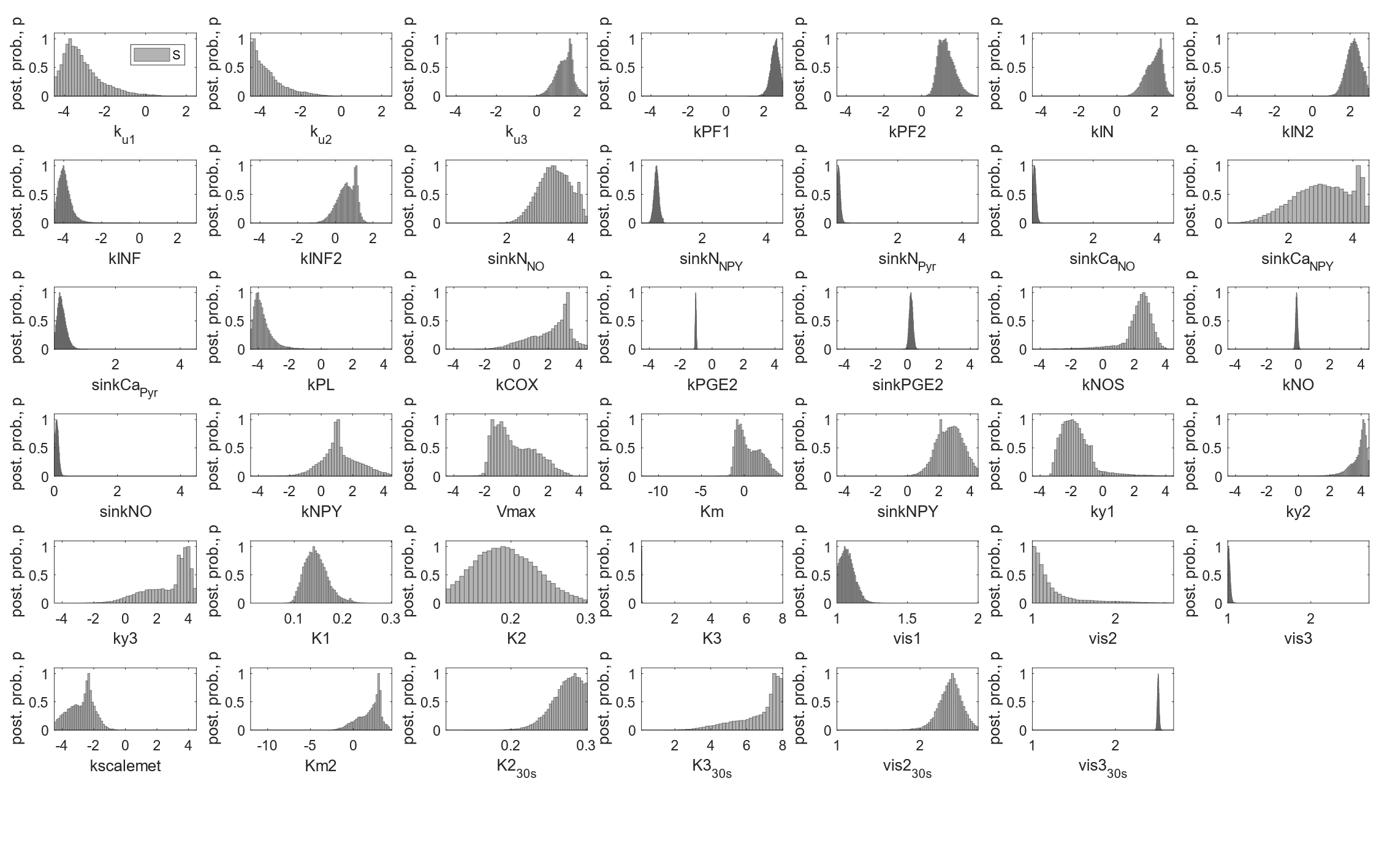

Supplement: S3 Fig — Posterior probability profile (y-axis) for each estimated model parameter (x-axis, log10 space) for the model estimated to data presented by Drew et al. [46], allowing the viscoelasticity and stiffness coefficients of the capillary and venous compartment change between 10 and 30 s stimulation. (TIF) [file pcbi.1010818.s005.tif]

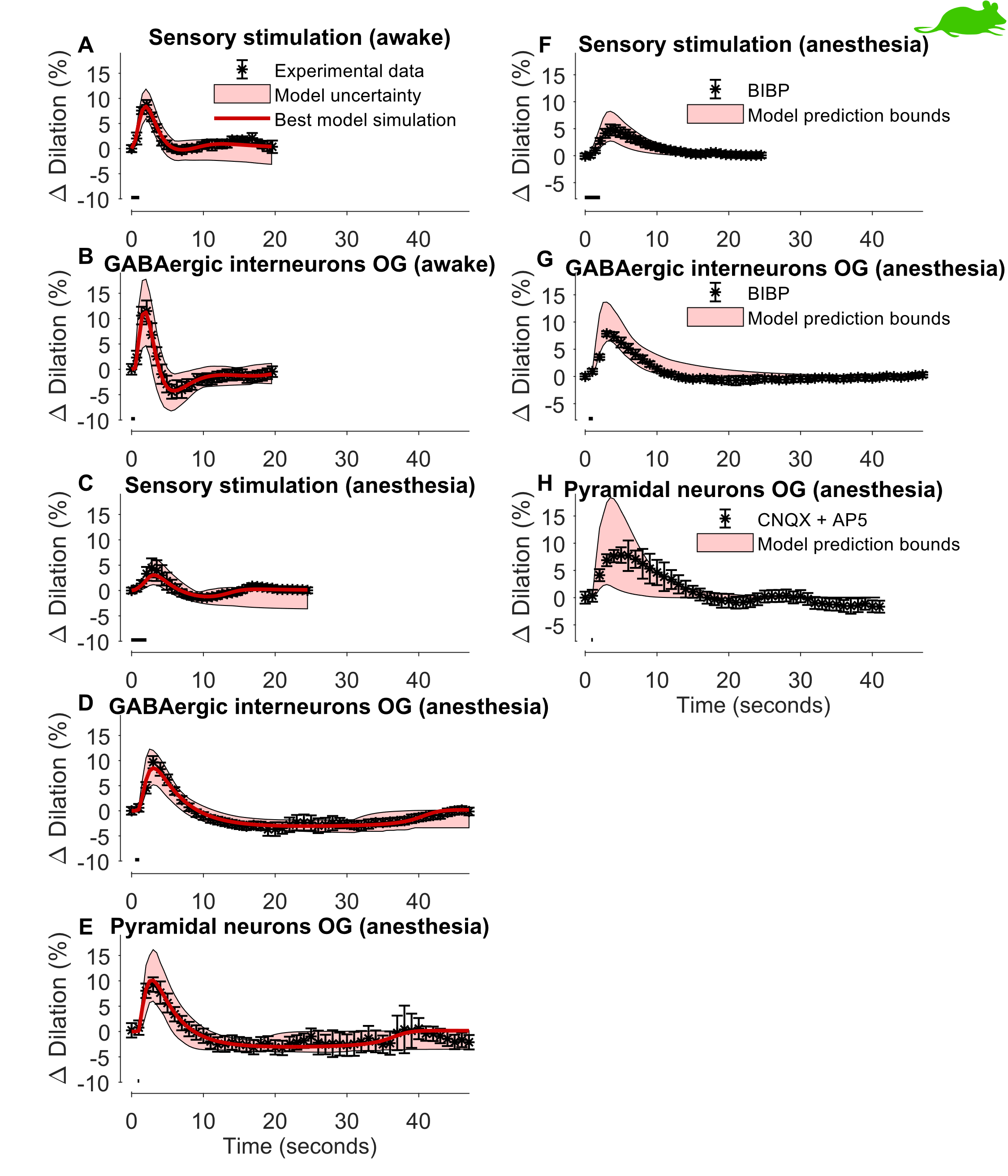

Supplement: S4 Fig — A-E: Model evaluation to arteriolar response data from awake (A, B) and anesthetized animals (C–E) for both optogenetic (OG) (B, D, E) and sensory (A, C) stimuli. F-H: Model predictions to drug perturbed arteriolar response data during anesthesia condition. OG stimulation (G, H) and sensory stimulation (F), during the presence of the NPY receptor Y1 antagonist BIBP (F, G) and glutamatergic signaling blockers AP5 and CNQX (H). For each graph, the best-estimated model simulation (solid red line, A-E only) paired with model uncertainty (red shaded areas) compared to experimental data (black symbols, error bars depicting standard error of the mean). The stimulation length is indicated by the black bar in the lower left portion of each graph. Experimental data originates from Uhlirova et al. [47]. (TIF) [file pcbi.1010818.s006.tif]

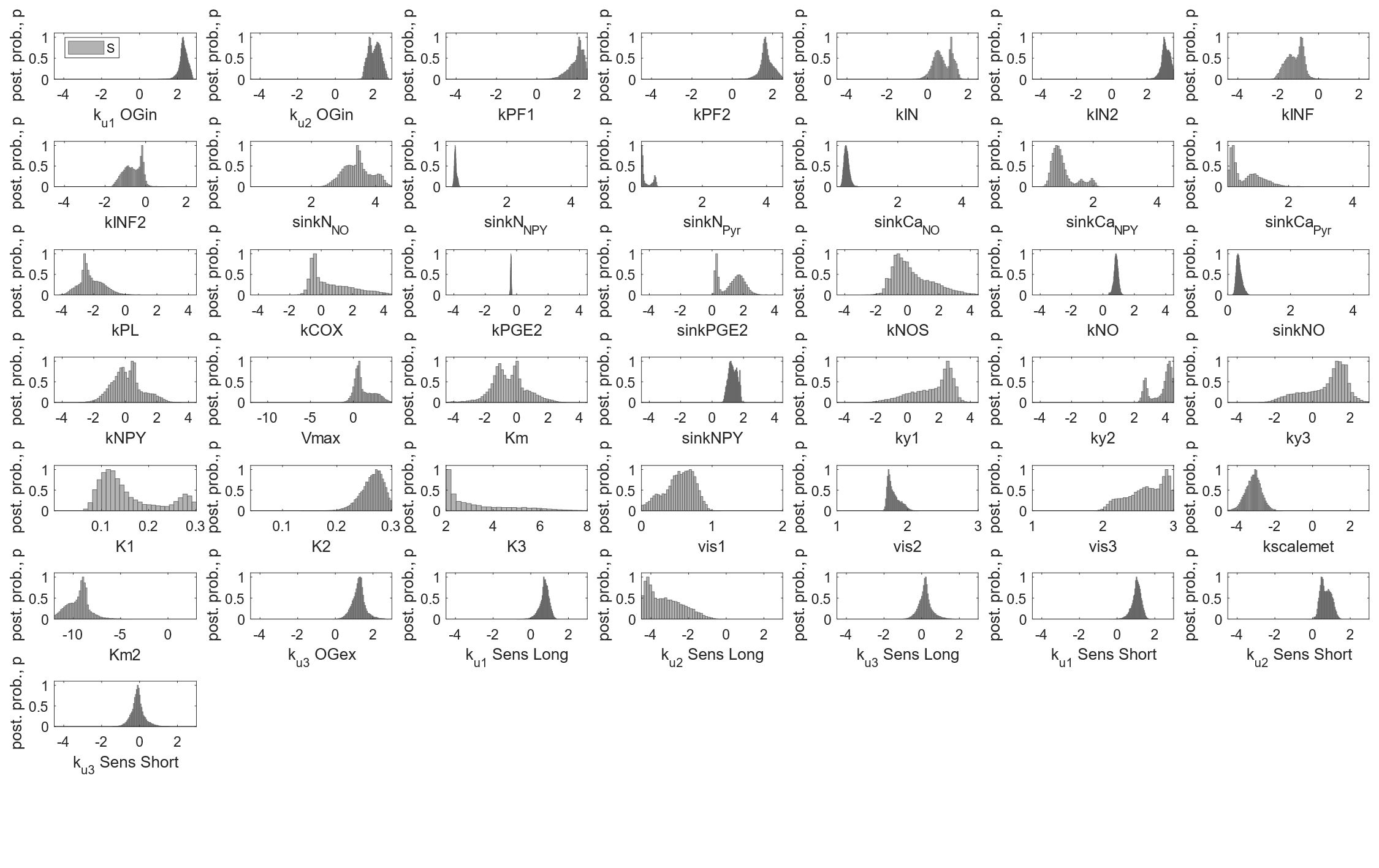

Supplement: S5 Fig — Posterior probability profile (y-axis) for each estimated model parameter (x-axis, log10 space) for the model estimated to data presented by Desjardins et al. [48]. The affix S represents the parameter value for sensory stimulation and is further subdivided into short and long sensory stimulation. (TIF) [file pcbi.1010818.s007.tif]

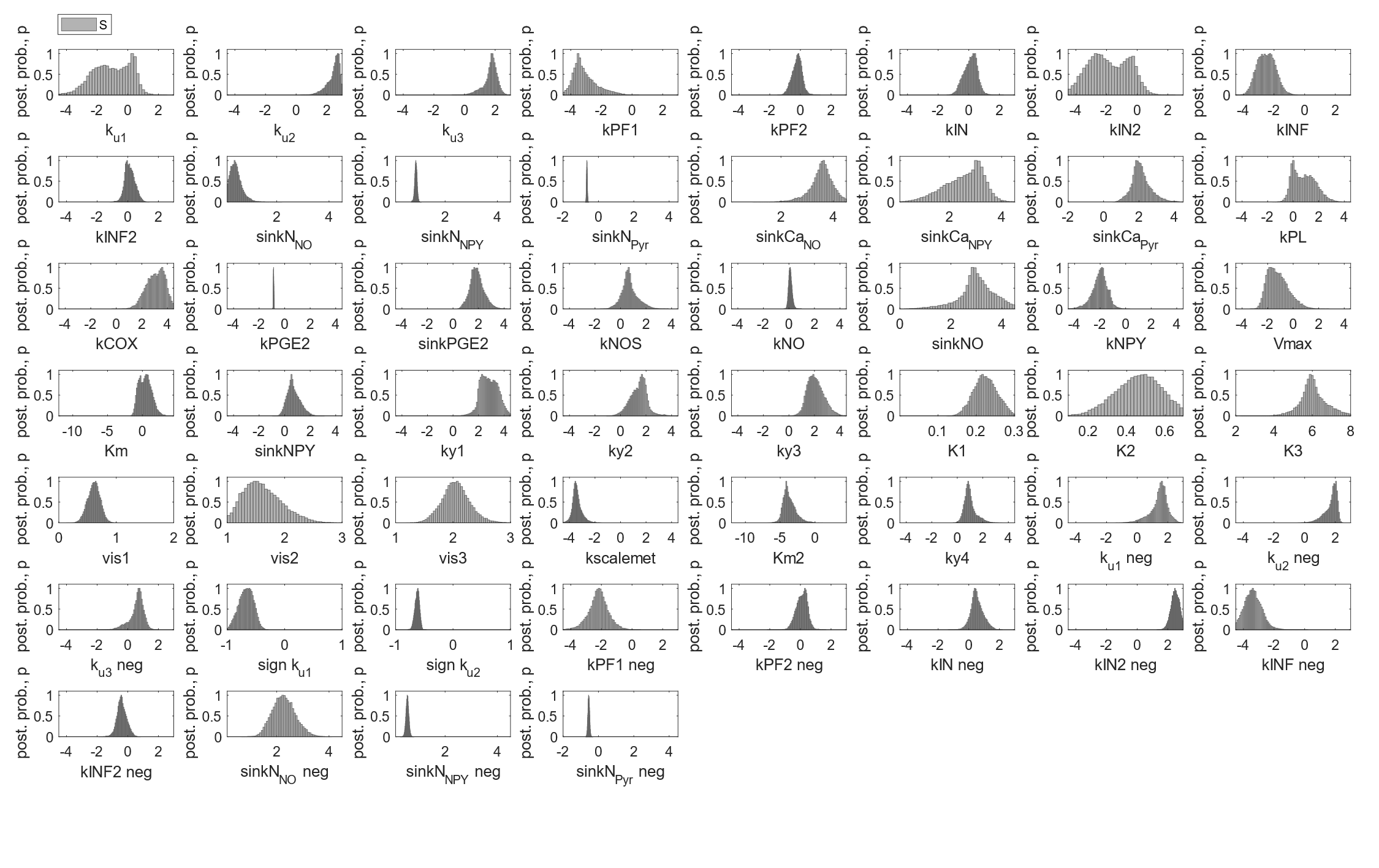

Supplement: S6 Fig — Posterior probability profile (y-axis) for each estimated model parameter (x-axis, log10 space) for the model estimated to data presented by Shmuel et al. [10]. The affix neg represents the parameter values for the negative response. (TIF) [file pcbi.1010818.s008.tif]

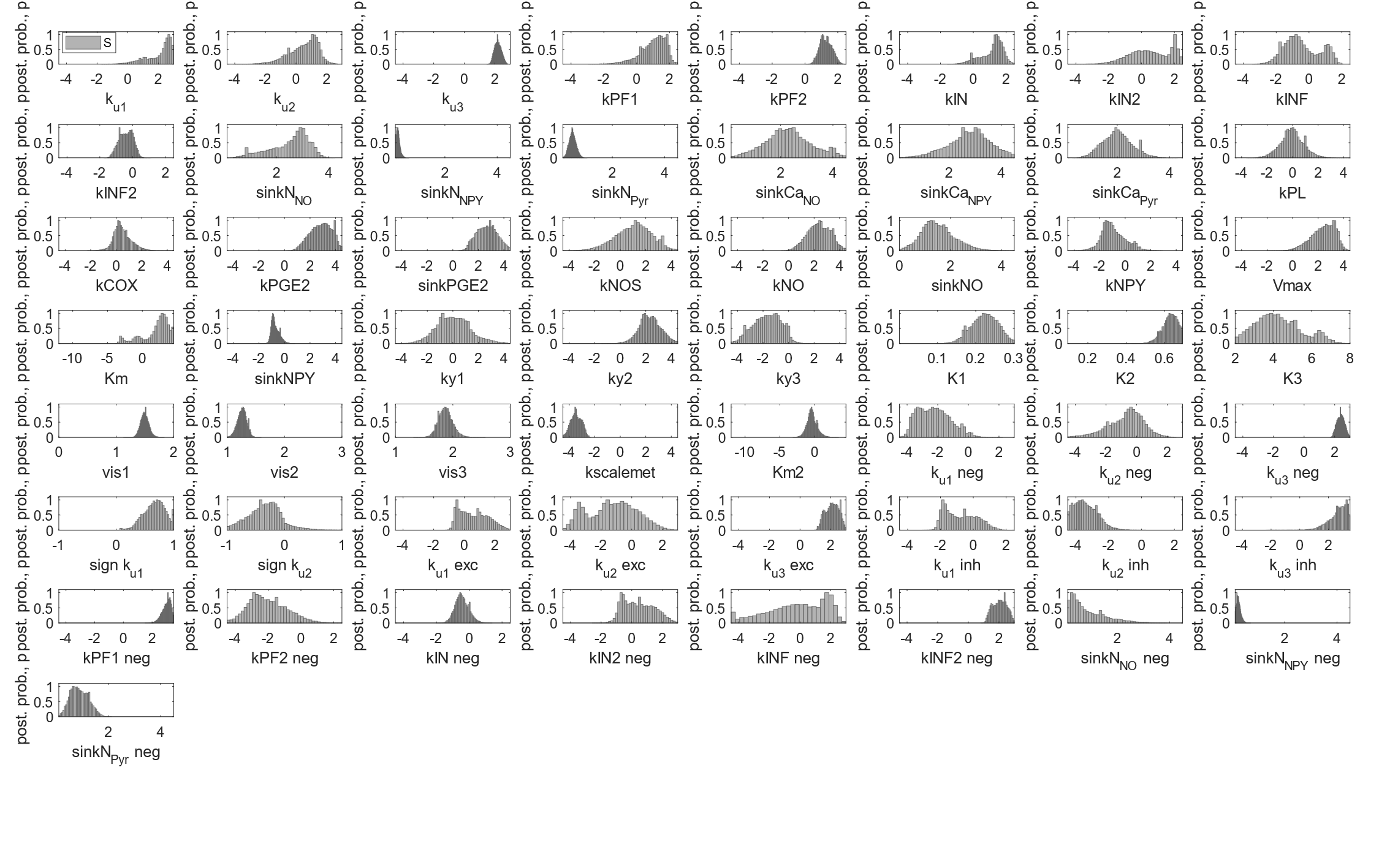

Supplement: S7 Fig — Posterior probability profile (y-axis) for each estimated model parameter (x-axis, log10 space) for the model estimated to data presented by Huber et al. [49]. The affix neg represents the parameter values for the negative response, exc represents the parameter values for the excitatory response, and inh represents the parameter values for the inhibitory response. (TIF) [file pcbi.1010818.s009.tif]
