# Supplementary material for: A quantitative model for human neurovascular coupling with translated mechanisms from animals
Source: PLoS Comput Biol. 2023 Jan 6;19(1):e1010818. doi: 10.1371/journal.pcbi.1010818 (PMC9821752; doi:10.1371/journal.pcbi.1010818)
Supplement: Attachment — Submitted filename: Response to reviewers.docx [file pcbi.1010818.s015.docx]

Reviewer's Responses to Questions

**Comments to the Authors:
Please note here if the review is uploaded as an attachment.**

Reviewer #1: This is a very interesting and comprehensive paper on the neurovascular coupling, the means by which blood flow is regulated throughout the brain. A mechanistic understanding of the NVC is important because functional neuroimaging uses hemodynamic changes as a proxy for neural activity.

In general, I think this paper is a good fit for PLOS Computational Biology, and it seems to be innovative. However, I found several aspects of the paper very unclear and would suggest clarification before publication.

Reply: We kindly thank the reviewer for their interest in our work and their feedback on how to further improve the article. We have made our best efforts to clarify the details that have been pointed out and further made an overall readthrough of the paper to look for more unclarities. We hope that our revision is to the satisfaction of the reviewer and that the details of the work have been sufficiently clarified.

1. I believe that each of the levels (neural response, hemodynamics, and BOLD response) are decoupled - that is, for instance, that the neural response affects the hemodynamics but not vice versa. If this is the case, that should be clarified; Fig. 2 is trying to mimic anatomy in some sense and is not depicting the modeling accurately. Additionally, in several of the schematics, there are arrows pointing to nothing, which makes the interactions unclear.

**Reply:** the reviewer has correctly interpreted that the different levels (neural response, hemodynamics, and BOLD response) of the models are decoupled and do not have a feedback system implemented. This feedforward, rather than feedback, architecture is in fact an intentional feature, which we demonstrated as being the correct alternative in a previous paper Lundengård et al 2016 (Doi: [10.1371/journal.pcbi.1004971](https://doi.org/10.1371/journal.pcbi.1004971)). We have now clarified this in relation to Figure 2. With this said, there is some further refining of the model that could be done in the future, which would add more cross-talk and feedback regulations. For instance, while the vascular response to neuronal activity seems to be driven by a feedforward, rather than a feedback, system, there is evidence for a control of vasculature also from metabolic sensing; if one e.g. lowers the oxygen levels or increases the carbon dioxide levels, this will lead to an increase in blood flow (Jaeger et al, DOI: <https://doi.org/10.1161/01.STR.0000257964.65743.99>; Carrera et al, DOI: <https://doi.org/10.1001/archneurol.2010.43>; Iadecola, DOI: <https://doi.org/10.1016/J.NEURON.2017.07.030>).

Regarding Fig 2. We have tried to fuse anatomy and a schematic model representation to give correlate our modeling thoughts to the anatomy. The arrows in question, which point to nothing, are indicating substance breakdown or dissociation, or blood flow between the different compartments (artery, capillary, venule). Reasonably, this is confusing when fused with the anatomical representation and we have added a clarification that the arrow in question now points toward the “empty entity” to indicate their function. This is also added to the figure text, on line 198. We would argue that the arrows are worth keeping since they indicate that the model handles the breakdown of substances and that it isn’t a continuation buildup. This means that the new and updated version of Fig 2 should be a one-to-one correspondence with the actual equations.

2. In the discussion, the authors mention that no existing model can describe "pharmacological perturbations including the effect of anesthetics", followed by a sentence that implies that this model can describe all of the aforementioned behaviors simultaneously. I did not see any information on anesthetics, so the statement seems misleading to me. This should be clarified.

**Reply:** It seems indeed like our previous formulation was clumsy; we thank the reviewer for catching this. As our model is an extension of our previous works, Sten et.al. 2020 (Doi: [10.1016/j.neuroimage.2020.116827](https://doi.org/10.1016/j.neuroimage.2020.116827) ), which were able to describe “pharmacological perturbations including the effect of anesthetics” and how these affected the blood vessel response. Our new model has inherited these abilities since it was built upon the previous work. Although we do not show these abilities in the main manuscript, we show that this ability is preserved in the supplementary material. We have clarified this and added a reference to the section in the supplementary materials where this is shown; these changes can be found on line 535.

3. The second limitation mentions the parameters used for different experiments and attempts to explain which were preserved or held constant and which were changed where. It is extremely unclear, and not helped by the fact that all the parameters are only shown in the supplementary information. I believe this could be conveyed more clearly with a table or perhaps a figure.

**Reply:** We recognize that our attempted explanation was insufficient, and we thank the reviewer for bringing this up. We have attempted to change as little of the model structure between each study as possible, however, the difference between the nature of the experiments requires us to change some things. More specifically, we have added some further explanations in the text, lines 674-679, and a new table (lines 688-688), as per the reviewer’s suggestion, to clarify the model design. In the Drew study no parameter changes (although the LFP parameter is not present) for figure 3-4. For the Desjardins study (Fig 5) the stimulus scaling parameter is allowed to change (k_u1-3_) since the experimental setting changes. For the optogenetic (OG) experimental setups all k_u_ parameters are not present since not all cell types are stimulated. This results in 4 sets of k_u_ parameters; OG inhibitory has k_u1_ and k_u2_; OG excitatory has k_u3_, Sensory long has k_u1-3_, and Sensory short has an additional set of k_u1-3_. Similarly, the different experimental settings in the Shmuel and Huber studies require us to change some parameters. The nature of the positive and negative stimulation paradigms results in a response in different areas of the brain. Therefore, we have allowed for the stimulus scaling parameters, neuronal signaling parameters, and the elimination rate parameters to differ in the Schmuel study. In The Huber study, the stimulus scaling parameters and neuronal signaling parameters are allowed to change between the experimental setups. However, the neuronal signaling parameters are kept the same within the positive and excitatory experimental setups, and the negative and inhibitory experimental setups. Again, we have strived for as little change as possible, but the nature of different species and experimental setups required some alternations to our model. We hope that our changes make it more clear where the changes are implemented.

4. The readability of the paper was severely impacted by embedding figure captions but not figures. As PLOS's guidelines for submissions allow this, please include a copy that has figures and captions in the text.

**Reply:** We thank the reviewer for the feedback and now submit such a version of the manuscript in this new submission.

5. The timing described in lines 234-236 is very confusing.

**Reply:** we thank the reviewer for pointing this out, our previous explanation was a very roundabout way to describe that we do not consider the train of air puffs as a stimulus (if we would, the stimulus would be a train of square pulses with duration 20 ms, a rest period of 105ms, and with a frequency of 8Hz). Rather, we choose to constantly apply the stimulation for the whole duration i.e. 125ms, 10 s, and 30 s for the 3 different experimental setups. We have altered lines 233-236 to clarify this.

6. The paper includes too many acronyms and uses them inconsistently - for instance, optogenetics is (rightfully!) used most of the time in the text, and only used as an abbreviation in the figure. Please consider replacing some acronyms (CBF, CBV, OG, etc.) with the full words in the text. Also, MCMC is used without definition.

**Reply:** We have followed the reviewer's advice and limited our use of acronyms in the text. Multiple acronyms are now completely removed from the text (OG, HbT, HbO, HbR, Hb, GLM, SPM, MRS) and we hope that the text now is more easily read. We also thank the reviewer for pointing out that MCMC was used without being defined; this is corrected now.

7. Relatedly: define all acronyms in figures. For instance, in Fig. 2, I believe u, f_i, and V_i are not defined.

**Reply:** We thank the reviewer for their attention to detail and have revised our explanation of the acronyms used in our figures. This change is available on lines 210-223.

8. The figures would benefit from some careful thought about design. A few examples - the "brown" in line 208 looks like red. Fig. 1C would be easier to understand if colors were used in addition to text. The legend in Fig. 7 is not even in order for each subplot. Also, I think the same colors are used for CBV and CBF.

**Reply:** we agree with the reviewer that our images are comprehensive and thus, the graphical design is important to convey the results to the reader. Considering the given advice, we have therefore implemented the following changes.

- We have changed the gradient of the “brown” color in Fig 2 to be more distinguishable from the red.
- Fig 1C has been updated to guide the reader through the abilities of the different models using colors (green - yes, red – no, yellow – partly), see lines 99-100.
- Regarding the legend in Fig 7, we have chosen to not order the colors directly in the order of the subplot. The reason for this, we argue, is that the grouping of the different symbols is more important. We think that the reader is better assisted by getting an overview of which variables have a simulation, experimental data, and model uncertainty (which are the first 5 lines in the legend), and thereafter the variable without a simulation is listed. These two subgroups are ordered by the subplots but due to our choice of ordering them this way, we end up with the arteriolar and venous CBV (Fig 7B-C) being presented after the annotations from fig 7D in the legend. Although unfortunate, we believe this is a better approach to building the legend in this case.
- The reviewer is fully correct in that CBF and CBV use the same color in Fig 7A and 7E. The reason behind this is simply that we have tried to remove some colors. Since we have tried to color-code all the experimental data throughout the article (for the reader to easily recognize the reoccurring variables), we end up with quite a lot of colors. Since CBV and CBF, in this case, are collected during the same experimental setup and measure the combined changes over the vessels (arteries, capillaries, and venules), we think that it is justified to group these two variables and use the same color. Partly to eliminate an additional color, partly since they are similar entities.

We hope that these corrections are in line with what the reviewer had in mind and that the figures better convey their messages.

Reviewer #2: The authors develop a model to couple i) neuron activity as a set of ODEs, ii) release of vasodilation/vasoconstriction molecules downstream of neural activity as a set of ODEs representing relevant reactions, iii) blood flow from a lumped model akin to electrical circuits, iv) oxygen metabolism downstream of blood flow. The model builds off previous work by connecting the different model modules from prior work. To calibrate the model the authors follow a Bayesian approach to explain a comprehensive dataset. The calibration of the model provides insights into the unobserved model signaling dynamics from the observed blood flow and oxygen observed data. While the model setup is comprehensive and data from the literature is also comprehensive, I do have some major comments:

**Reply**: We thank the reviewer for taking interest in our work and for the advice for making the work better. We have gone over our manus to clarify parts that caused confusion and tried to more clary describe our reasoning more. For instance, in line with the reviewer’s advice, we have added tables in the supplementary materials with the upper and lower bounds for the parameter posteriors. More details follow below.

[1] Line 185: cross reference to validation data seems wrong. The same cross reference is repeated later in the text. Please check other cross references in case I missed other ones, but the validation ones are crucial which is why I noticed this.

**Reply:** we thank the reviewer for the great attention to detail; there were indeed incorrect cross-references. We have corrected these throughout the manuscript in our revision.

[2] Line 233: How was the stimulus prescribed in the model?

**Reply:** As our previous description was unclear and poorly written, we have revised this description to better describe how the experimental setting was translated to the model. We choose to not consider the on/off dynamics of the air-puff train and apply the stimulus as a constant on-signal throughout the experimental stimulus phase (i.e., stimulus duration of 125ms, 10 s, and 30 s for the different experimental settings). The updated description is located on lines 233-236.

[3] Line 242: where all the model parameters fitted? The authors seem to be using a Bayesian approach which makes me think that distributions of the parameter posterior are estimated. That would be great, much better than standard fitting, but I am definitely curious how loose/tight were the priors and whether the posteriors are reasonable. The authors should refer the reader to these statistics in this section, even if the statistics of the Bayesian fitting are reported in the Supplement. I understand some of the priors are defined in the methods, but a cross reference to the posterior traces in the Results would be great.

**Reply:** Our modeling approach is built upon hypothesis testing, and the parameters were indeed estimated using Bayesian methods. In short, we establish a hypothesis, from knowledge of biology, of the system and evaluate these hypotheses against experimental data. The hypothesis might (most often is) be revised and altered until we end up with a model structure that can describe the experimental data. In this phase, we do optimize our parameters. In this work, all parameters have been optimized, apart from the ones with values derived from the literature (i.e., blood pressure constants). The next step would be to add more experimental data to further test if our hypothesis and model structure are robust, which repeats the hypothesis testing process. Once a model structure sees robust enough, we additionally test it using validation data. This is data that the model has not had access to during training and to accurately describe this data the model structure needs to be true enough compared to the real system and we can conclude that our hypothesis is one step closer to describing the real system. After this, we can additionally estimate our parameter posteriors to deduct how well defined or model is (how wide the range of values that the model parameters can be and still provide an acceptable fit). We have done this by performing Markov Chain Monte Carlo sampling and collecting the range of all parameters which pass chi-square tests for fitting the experimental data. As per usual with MCMC parameter sampling, we start the sampling at the optimum obtained from the initial optimization. We use boundaries for the parameters, and we use qualitative constraints to preserve features from lower animal systems to higher; no other prior was used. As the reviewer has pointed out, we had not explicitly stated the parameter boundaries used during these estimations. We have revised this and added the upper and lower bounds for the parameters in the supplementary materials and added cross-references to these tables. This specific data set is referenced on lines 242-244.

[4] Figure 4: What are u_1, u_2, u_3? Are these the inputs to the simulation? This is related to my comment [2], it is not clear what drives the simulation

**Reply:** The reviewer brings forth a good remark that we had missed specifying the role of u_1, u_2, and u_3 in figure 4. As the reviewer has suggested u (1,2, and 3) represent electrical stimulation needed to increase the levels of intracellular calcium in the neurons and is indeed the stimulus for the model. The reason for the different notations (1,2,3) is that while they can be the same in sensory stimulation, optogenetic stimulations only activate specific neuron populations (depending on the experimental setting) and we need thus to distinguish the stimulation of the different neuron types. This is better described in our revised manus, both in figure 4 lines 294-295 and figure 2 on line 200.

[5] Figure 4 E-G, why are NO values negative? I understand these are arbitrary units but in principle the three variables shown in these plots are concentrations and are expected to be positive. Since the Methods is left for later on in the manuscript, a note as to why we can expect negative values for these ‘concentrations’ should be added.

**Reply:** As the reviewer has noted we have failed to clarify the implications of the negative values. As the model needs to be calibrated into a “steady state” to be responsive to only the applied stimulus, we express the vasoactive effect on the vascular smooth muscle cells as the difference between the simulated concentration of the vasoactive substance, i.e., nitric oxide (NO), and the steady state value of NO. This is described in equation 8 and can be described as follows NO_SMC = NO(t) – NO(0) (scaled with a parameter). However, we missed to state this on the y-label and have added a ‘delta’ to highlight that it is the relative differences that are illustrated. This is also highlighted in the figure text in lines 306-308.

[6] Line 348: Were the input parameters manually adjusted or all parameters, including inputs, determined with a Bayesian calibration? And if the inputs were part of the optimization, were the priors fairly tight? Do the posteriors for the inputs (in case they are part of the Bayesian framework) make sense?

**Reply:** the experimental inputs (i.e., stimulation length) were set to the same values as the experimental paradigm stated in the different studies and were not part of the optimization. As our stimulation is a square pulse with a set amplitude, $\left\{ \begin{aligned} 1, if 0<t<t\_stimend \\ 0, if t>t\_stimend \end{aligned} \right\}$, we choose to include stimulus scaling parameters k_u1-3_ since we cannot interpret how strongly different types of stimulations (air puffs, optogenetics, visual stimulation, etc.) affect the neurons and how they relate to each other. The priors for k_u1-3_ are now available in Table S1 (supplementary materials). We would like to argue that the posteriors do make sense. They are somewhat wide, however, as the k_u1-3_ parameters interplay with the KPF, KIN, KINF, and sinkN_NO, NPY, Pyr_ parameters to decide the neuronal activity for the neurons and this interplay allows for various combinations of parameter values. As we have no literature to restrict these actions, we have allowed the posteriors to be fitted freely between our boundaries. The result is that the posteriors mostly have a defined interval smaller than the allowed posterior space and they are well-defined within these regions; this thus makes the posteriors reasonable.

[7] Line 373: Different model of parameters for Figure 5 and Figure 4. Is this because the inputs were fitted for Figure 5 but not for Figure 4? Or is it because different parts of the model were trained in Figures 4 and 5.

**Reply:** We realize that our explanation for the different parameters was not clearly stated and have now added a table to improve this, see lines 688-698. The reason for the different parameter values is that the studies were performed independently from each other and with different experimental settings. In the case of Drew, air puff stimulation was utilized, and in the case of Desjardins, air puff stimulation and optogenetic stimulation were used. Since the optogenetic stimulation does not target all neurons k_u3_ is excluded from the inhibitory optogenetic stimulation and k_u1-2_ is excluded from the excitatory optogenetic stimulation. Otherwise, the same parameters are present in the model structure, but the values differ between the studies. We have chosen to not fit the studies together since we have focused on our idea of fitting the data using the translated mechanisms and transferring the qualitative behavior between the studies while allowing different parameter values. This is done since the species and experimental setups do differ from each other; it would thus be unreasonable to require the model to have the same parameters in these different settings.

[8] Related to my previous comment, between Figures 4 and 5, were the same parameters trained in both cases, and if that’s the case, then how different are the posteriors? They are both for rodent data, so one would expect the posteriors to be similar. Is that the case? Cross reference to comparisons of the posteriors is needed.

**Reply:** As the reviewer points out the data from the Drew and Desjardins study are both collected from mice. The experimental data from the two studies were fitted independently by the model, resulting in different parameter profiles for the two model fits (see added table 1 for a graphical representation of how the parameters were fitted between the studies). Since both of the studies were carried out on mice, it would be natural to compare the posteriors between them. We have added such an analysis of the posterior probability profiles, between Figure 3/4 and Figure 5, in supplementary materials section 1.7.1 and added a cross-reference to this analysis on lines 418-419. In short, apart from the stimulus scaling parameters, k_u1-3_ (which are expected to differ), the neuronal signaling parameters, elimination rate parameters, and intracellular signaling parameters have similar profiles between the independently fitted experimental data. This suggests that the model finds a similar behavior of the neuron module that is identified in both data sets. The viscoelasticity and stiffness coefficients have some profiles matching quite well (K1, vis1, vis2). Overall, it seems that the model could have described these data with one parameter set. Moreover, the model identifies similar profiles between the different independently fitted studies – indicating that the model finds a similar behavior in both data sets.

[9] Line 418: How are the positive and negative inputs prescribed in the model? I understand more details are available in the Methods, but a simple clarification in the results would be helpful. For example, if N_NO neurons are activated in the model to simulate one of the cases then it can be stated without going into all the details of the simulation setup. It is difficult to make sense of the outputs shown in figures without having a very brief reminder of what the input is for the simulation.

**Reply:** In line with the suggestion made by the reviewer we have added a short description of how the stimulus was applied to the model. In the case of positive stimulation, a square pulse is applied as stimulation. In the case of the negative stimulation, we included sign parameters for the model to interpret if the stimulation should act positively or negatively on the NO and NPY neurons, as this wasn’t known from the literature (pyramidal neurons are known to have a decreased activity from a negative visual task and were thus set to have a negative square pulse). In the model posteriors (Figure S6) one can see that sign k_u1_ and sign k_u2_ lie in the range -1 to 0 and thus the stimulus is interpreted as a negative square pulse $\binom{-1, if 0<t\leq t\_stimend}{0, if t>t\_stimend}$ for all three neuron types.

[10] Line 422: why are the number of parameters 52 for the macaque data in Figure 6?

**Reply:** In line with previous comments, we refer the reviewer to our new Table 1 at lines 688-698, for a better overview of how the parameters were handled, and why there are different numbers of parameters for the different studies. With the two different visual stimulation paradigms, activating different areas of the brain, it was required to have different sets of parameters governing the neuronal activity (stimulus scaling parameters (ku1-3), neuronal signaling parameters (KPF, KIN, and KINF), and elimination rate parameters (sink_NO, NPY, PYR_). While the rest of the parameters were the same between the different experimental setups, the 2 different sets of neuronal activity parameters, 1 set of intracellular and vascular control parameters, and the addition of ky4 (LFP scaling parameter) and 2 sign parameters (which is present in the negative experimental set up) adds up to a total of 52 parameters. Note that the two sign parameters are passed as constant to the model and simply control if the stimulus should have a positive or negative impact on the N_NO and N_NPY state during the negative stimulation. We choose to allow these to be decided by the optimization process as we could not guarantee the effect of the negative stimulation on the neurons. In practice, we could treat these 2 parameters as constants after deciding the effect on the neurons, which would reduce the number of parameters to 50.

[11] Going back to comment [1], so far only Figure 5J has validation data, all other plots seem to be results from model training. This is fine, but then the notion of ‘validation’ data is misleading. What do the authors mean by ‘validation’?

**Reply:** The reviewer is correct that Figure 5J is the first occasion of validation data. Apart from this, Figures 7B, C, and E also show instances of what we call validation data. Validation data is a set of data, from the same study, that we have excluded from the model training phase to validate that the model behavior is robust. In the case of the Desjardins study (Figure 5), the experimental data in Fig 5A-F were used in the model training phase. After this phase is completed, we allow the model to predict how a BOLD response would look under the same experimental setting as the collected experimental data (20 sec optogenetic excitatory stimulation, Figure 5J). As can be seen, the model predicts a good BOLD response with the parameter posteriors achieved during the model training phase. This is what we mean with “validation”, that the model successfully predicts the behavior of experimental (validation) data that it has not observed previously. This validation argues for a robust model structure and is a method for us to test the logic that the model is built upon and that the model training did not result in parameter posteriors that only can handle the training data. However, we do acknowledge that there are different interpretations of the word validation, where some people use the word only in more rigorous settings, where all aspects of the model have been validated; we use the word to describe a successful prediction of some data not used for estimating the parameters.

[12] Figure 7: How many parameters were trained for the human case? In previous Figures 4,5,6 there was a different number of parameters per figure. Now I’m curious how many parameters are there for Figure 7. Also, I’m still curious about the posteriors, and whether posteriors from different re-training align with each other.

**Reply:** In line with previous comments, we refer the reviewer to our new table 1 at lines 688-698, for a graphical representation of how the parameters were handled. In the human case, we have multiple different visual experimental setups, a positive stimulation, a negative stimulation, an excitatory stimulation, and an inhibitory stimulation. Although different experimental setups, the positive and excitatory setup, and the negative and inhibitory setup are similar to each other. We have therefore preserved the neuronal signaling parameters within these two groups. However, as there are four different experimental setups, we have allowed the model to have a unique set of stimulus scaling parameters for each experimental setup. This results in 4 sets of experimental stimulus parameters, 2 sets of neuronal signaling parameters (KPF, KIN, KINF, sink_NO, NPY, PYR_), 1 set of intracellular signaling, vascular control, and sign parameters. This collection of parameters results in 57 parameters. Like the case for Figure 6, 2 sign parameters were included to decide the effect of the negative stimulation on the neurons. These could be treated as constants to reduce the number of parameters to 55.

[12] Line 492: When the authors say that they translated all mechanisms from rodent and macaque to the human case do they mean that after re-training independently to each data set, from rodents, to macaque, to human, the features of the inferred dynamics for the neural responses N_NO, N_Pyr, N_NPY align in all cases? Or was there actually something that the authors did to ‘transfer’ parameter information from one training to another? For instance, did the authors use the posteriors from training in Figure 4 as priors for Figure 5? If every training is done independently, then I’m not sure ‘translation’ is the most accurate word. Even in that case, the fact that the inferred dynamics for N_NO, N_Pyr, N_NPY and their contribution to vasodilation/vasoconstriction is preserved with the different data-sets is remarkable and very interesting.

**Reply:** The reviewer raises an important question of which answer would have a different interpretation of our work. We did indeed train the model to one data set at a time. To further clarify the process, we started with the data from the Drew study (Figure 3) and from the resulting posteriors we observed the behavior of the neurons' activity that resulted in the “double peak” behavior in the Drew data (Figure 4). From this behavior, we formulated *qualitative behaviors* (see section S2, equation S1-S6) that were essential in explaining the data from the Drew study. Following this, we moved on to the Desjardins study. In addition to the experimental data, the qualitative behavior identified in Figure 4 was also imposed on the model during the model training phase of the new data. The result is shown in Figure 5, where Fig 5G-I shows that the demands identified from the Drew study (section S2, equation S1-S6) hold for these new posteriors. Continuing, we formulated the hemoglobin behavior as demands (section S2.2) which were additionally imposed on the model training for the Shmuel study. From the Shumel study, the neuronal behavior during negative stimulation and the LFP behavior was identified and imposed on the model training of the human data. To summarize, four different iterations of model training were performed, one for each study, where additional qualitative demands were imposed on each consecutively model training phase. To clarify, the same model structure was used but four different posterior profiles were identified. This means that we use qualitative constraints on the simulations and not priors to the parameters themselves.

Our reasoning behind the choice of “translated” mechanisms comes from our process of formulating the qualitative demands from each study and imposing them on the following studies during their model training phases. As there is no, or very limited, invasive experimental data available on human subjects one could not find these behaviors from human studies. We argue therefore that these behaviors are “translated” from the respective animal study.

[13] Eq. 6a shows a very standard eq. for NO from which I don’t expect a negative NO value. Why are there negative NO values in the results?

**Reply:** The reviewer is correct that this equation will not generate any negative values for NO. However, the NO we show in figure 5,6,7 is NO_VSM which is the relative difference between NO and the value at t = 0 (NO(0)). What we see in Figure 5,6,7 is that after an initial spike of NO produced, the production is reduced below that of basal levels. The effect of NO on the vascular smooth muscle (VSM) cells is explained in equation 8.

[14] There are cross references to Section 7, I assume the authors refer to the now-Supplement.

**Reply:** We thank the reviewer for catching this typo, it is now corrected to section 6 since it was pointing toward the Methods section.

[15] Regarding a previous comment on prior and posterior reporting and comparison between posteriors. The Supplement shows the different posteriors. How close are the posteriors between independent trainings from Figures 4-7 should definitely be done. Also, a table with the priors is needed.

**Reply:** A table with the priors, in which the boundaries for the parameters are included, is now added in the supplementary, Table S5. We have also added an analysis of the posteriors from Fig 3-7 in supplementary materials, section 1.7.2, and added cross references and lines 474 and 530. Interestingly, one can observe the neuronal signaling parameters, KPF, KIN, and KINF, and elimination rate parameters, Sink_NO, NPY, PYR_ are quite similar over the four different profiles. The human and monkey profiles generally have a wider range of probability. This is potentially due to the experimental setting, the visual stimulation, which arguably is less intensive compared to the stimulus of the mice, vibrissae, and optogenetic stimulation. The lower intensity could result in a wider range of parameter values being able to describe the observed behavior. As the only information that is carried over between the profile analysis is the qualitative demands that we identify from each study, it seems that the demands hold enough information for the model to identify similar behavior, and posterior profiles, for the four studies. This also argues for the behavior of the species being similar enough for the model to be able to describe them with similar behavior.
